# A generalized phase resetting method for phase-locked modes prediction

**DOI:** 10.1371/journal.pone.0174304

**Published:** 2017-03-21

**Authors:** Sorinel A. Oprisan, Dave I. Austin

**Affiliations:** Department of Physics and Astronomy, College of Charleston, Charleston, SC, United States of America; McGill University Department of Physiology, CANADA

## Abstract

We derived analytically and checked numerically a set of novel conditions for the existence and the stability of phase-locked modes in a biologically relevant master-slave neural network with a dynamic feedback loop. Since neural oscillators even in the three-neuron network investigated here receive multiple inputs per cycle, we generalized the concept of phase resetting to accommodate multiple inputs per cycle. We proved that the phase resetting produced by two or more stimuli per cycle can be recursively computed from the traditional, single stimulus, phase resetting. We applied the newly derived generalized phase resetting definition to predicting the relative phase and the stability of a phase-locked mode that was experimentally observed in this type of master-slave network with a dynamic loop network.

## 1 Introduction

Oscillatory neural activity is ubiquitous and covers a wide spatial and temporal scale from single neural cells to whole brain regions and from milliseconds to days. Neural oscillations are believed to be relevant for a wide range of brain activities from sensory information processing to consciousness [1]. It is believed that the phase of low frequency theta oscillations (4-8 Hz) drives the pyramidal cells and is used for information processing in the hippocampus [2–4]. Visual stimuli binding is believed to be related to the phase resetting of the fast frequency gamma band (30-70 Hz) [5]. Positive phase correlations between the theta rhythm and the amplitude of gamma oscillations were found during visual stimuli processing and learning [1, 6, 7] and during fear-related information processing [8, 9]. Theta rhythm resetting also drives cognitive processes [10]. Theoretical studies suggested that phase resetting could explain cross-frequency phase-locking of gamma rhythm within a theta cycle [11], which is the hallmark of successful memory retrieval [12, 13]. The phase of neural oscillations is also used to bridge a much wider frequency range from slow theta rhythms of large neural networks, such as those in the hippocampus, up to the individual fast spiking neurons used for speech decoding [14]. It was found that speech resets background (rest) oscillatory activity in specific frequency domains corresponding to the sampling rates optimal for phonemic and syllabic sampling [14, 15]. Phase resetting is also critical in the functioning of suprachiasmatic nucleus that produces a stable circadian oscillation by light-induced resetting of endogeneous rhythm [16, 17]. It was also shown that single sensory stimulus [18, 19] and periodic train of inputs [20, 21] induce phase resetting in electroencephalograms, which manifest as event-related evoked potentials.

Most of neurobiologically inspired interval timing theories assume that neural oscillators and their relative phases could be used as internal clocks for biological rhythms [22]. It was experimentally and computationally found that noisy neural oscillators could produce accurate timing that also obeys scalar property, i.e. the temporal estimation error increases proportionally with the duration [23–25]. The attention mechanism phase resets neural oscillators and can produce either a stop or a delay in conditioned stimuli timing with intrudes such as gaps [26] or fear stimuli [27].

Recent optogentic experiments shown that the steady gamma rhythm of medial prefrontal cortex can be reset and entrained by light stimuli and modulated by amphetamines [28]. Delay embedding reconstruction of the phase space gave a low dimensional attractor suggesting a phase coupled model of medial prefrontal cortex that is reset by light stimuli [29].

Unidirectional coupling between neural oscillators, i.e. a master-slave system, suggests the simplest possible synchronization mechanism that uses phase resetting to drive a neural population to a desired phase-locked firing pattern. Phase resetting methodology has been successfully used for predicting one-to-one entrainment in networks where the receiving population always follows the driving population [30–32]. It was recently shown that unidirectional coupling also allows for “anticipated synchronization” [33] in which the receiving population anticipates the states of the driving population [34]. It has been analytically proven and numerically verified that time-delayed feedback can force coupled dynamical systems onto a synchronization manifold that involves the future state of the drive system, i.e. “anticipating synchronization” [33]. Such a result is counterintuitive since the future evolution of the drive system is anticipated by the response system despite the unidirectional coupling. It has been suggested that delayed coupling in dynamical systems separated by some distance can still promote synchronization despite the slow signal transmission and the unidirectional coupling. The first anticipating synchronization study of excitable systems was done by Ciszak et al [35], followed by more recent behavioral-related investigations [36, 37]. Synaptic delay and synaptic plasticity was recently extensively investigated as potential control parameters that can lead to tunable delayed and anticipating synchronization in neural networks [38–40].

We investigated analytically and numerically a three-neuron master-slave system with a dynamic inhibitory loop that was previously shown experimentally to exhibit anticipating synchronization [41, 42]. The three-neuron network investigated here was shown to produce both delayed synchronization, in which pre-synaptic neuron fires a spike before post-synaptic neuron, and anticipating synchronization. It was argued that the delayed synchronization is a possible mechanism for spike-timing dependent plasticity [40], whereas anticipating synchronization could contribute to long term depression of synaptic couplings [40–42]. This study focuses on deriving analytic criteria for the existence and the stability of phase-locked modes in a three-neuron network that was found to generate both delayed and anticipated synchronization. For this purpose, we used the method of phase response curve (PRC) [32, 43–50]. The novelties of this study are (1) the generalization of PRC to multiple inputs per cycle and (2) the prediction of phase-locked modes in a neural network that is no longer limited to one-to-one firing patterns.

## 2 Phase response curve method

The phase response curve (PRC) method has been extensively used for predicting phase-locked modes in neural networks [51–54]. It assumes that the only effect of a stimulus is to reset the phase of the ongoing oscillation of a neuron. Traditionally, the PRC tabulates the transient change in the firing frequency of a neural oscillator in response to one external stimulus per cycle of oscillation. The term PRC has been used almost exclusively in regard to a **single stimulus** per cycle of neural oscillators. Recently, we suggested a generalization of the PRC that allowed us to account for the overall resetting when two or more inputs are delivered during the same cycle [55]. As a result, we expanded the PRC theory from the prediction of the traditional one-to-one phase-locked modes to arbitrary phase-locked firing patterns. Here we present the first quantitative application of such generalized PRC approach to a realistic neural network with a dynamic feedback loop.

**Fig 1:**
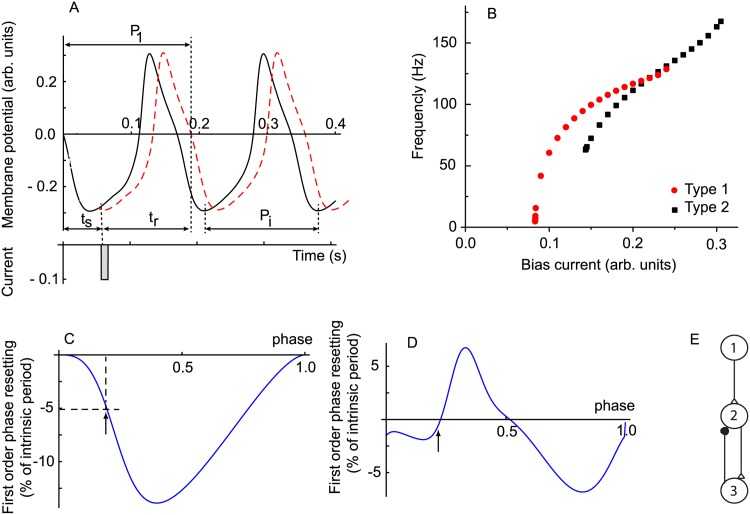
Typical PRCs for different classes of excitable cells. (**A**) The free running neural oscillator (continuous line) with an intrinsic period *P*_*i*_ is perturbed at stimulus time *t*_*s*_ by a brief current pulse (see shaded rectangle). As a result, the membrane potential is perturbed (dashed line) and the period of oscillation is transiently modified to *P*_1_, which induces a phase shift of all subsequent spikes. The time it takes a neuron to recover from a stimulus until it reaches the arbitrary zero phase reference again is called recovery time *t*_*r*_. Higher order PRCs measure the relative change in the firing period of the second and subsequent cycles (not shown). (**B**) Class I excitable cells can fire with arbitrarily low frequency by adjusting a bias current (solid circles), whereas class II excitable cells can only start firing at a minimum frequency (solid squares). The experimentally observed class I/class II distinction between neural oscillators translates in an (almost) one-to-one correspondence in type 1 (unimodal) PRCs (**C**) and, respectively, type 2 (bimodal) PRCs (**D**). The vertical arrow indicates a stimulus delivered at phase *φ* ≈ 0.2 that produces a 5% shortening of the intrinsic firing period in a type 1 (**C**) and 1% resetting in a type 2 (**D**) neural oscillator. The neural network for which we used the PRC to predict the phase-locked modes has three neurons: #1 is the pacemaker (master) of the network as it receives no feedback and drive the half-center formed with neurons #2 and #3; neuron #2 (slave) receives two inputs: one forward excitatory (open triangle) from the master neuron #1 and the other inhibitory (solid circle) from the interneuron #3; the interneuron #3 only receives one excitatory input (open triangle) from neuron #2 (slave) (**E**).

In the case of a single stimulus, the PRC measures the change of the free running period *P*_*i*_ of a neural oscillator to a new value *P*_1_ (see Fig 1A). The stimulus time *t*_*s*_ is measured from an arbitrary phase reference *φ* = 0. In our numerical simulations, the phase reference was the zero crossing of the membrane potential with a positive slope. The relative change in the duration of the current cycle, i.e. the cycle that contains the perturbation, with respect to the unperturbed duration *P*_*i*_ determines the first order PRC in response to a single stimulus (for detailed mathematical definitions see Appendix 1). As a result of the perturbation, the new firing period becomes *P*_1_ = *P*_*i*_(1 + *F*^(1)^(*φ*)), where *F*^(1)^ represents the relative shortening/lengthening of the intrinsic firing period *P*_*i*_ due to the stimulus applied at phase *φ* = *t*_*s*_/*P*_*i*_ [47, 48, 50].

A saddle-node bifurcation, which presents a continuous frequency versus bias current (f-I) curve that extends to arbitrarily low frequencies (see solid circles in Fig 1B) usually leads to a type 1 PRC that looks unimodal as in Fig 1C (although for counterexamples see [49, 56]). Fig 1C shows a typical type 1 PRC in response to a brief excitatory current perturbation that produces only phase advances (period shortening), i.e. negative resettings. A type 1 PRC looks unimodal and is often associated with a class I excitable cell, i.e. a cell that can produce stable oscillatory activity with arbitrarily low frequency [57, 58]. Usually, such excitable cells produce stable oscillations via a saddle node bifurcation on an invariant circle [59]. A type 2 PRC looks bimodal (see Fig 1D) and is often associated with a class II excitable cell [57, 58]. Class II oscillations usually emerge through a Hopf bifurcation [59] (see Fig 1B). As a side note, it was recently shown that type 1 (unimodal) PRCs do not always come from a class I excitable cell [56] and in fact all PRCs are bimodal with varying degrees [49, 50].

Close to the bifurcation point, accurate analytical formulas called normal forms describe the PRCs (see [57] and Appendix 1 for mathematical details), which we used in this study to get some analytical insights into the general behavior of the three-neuron network with a dynamic loop shown in Fig 1E.

The key assumption in generalizing the PRC method to multiple inputs per cycle was that the resetting induced by one stimulus takes effect “almost” instantaneously, i.e. before the arrival of the next stimulus [55]. Therefore, the effects of two stimuli applied during the same cycle are independent of each other. As a result, we used the single stimulus PRCs (*F*^(1)^) shown in Fig 1C and 1D to compute the phase resetting in response to two or more stimuli (see Appendix 1 for the detailed mathematical derivation of *F*^(2)^ and its generalization). Briefly, the first stimulus delivered at stimulus phase *φ*_*a*_ = *t*_*sa*_/*P*_*i*_ produces a transient change in the firing period to *P*_*a*_ = *P*_*i*_(1 + *F*^(1)^(*φ*_*a*_)). The second stimulus that arrives at a stimulus phase *φ*_*b*_ = *t*_*sb*_/*P*_*a*_ > *φ*_*a*_ further changes the firing period to *P*_*b*_ = *P*_*a*_(1 + *F*^(1)^(*φ*_*b*_)) (see Appendix 1). Combining the above effects of the two stimuli applied at phases *φ*_*a*_ and *φ*_*b*_, the new firing period *P*_*b*_ becomes *P*_*b*_ = *P*_*i*_(1 + *F*^(2)^(*φ*_*a*_, *φ*_*b*_)) (see Appendix 1 for a detailed mathematical derivation).

**Fig 2:**
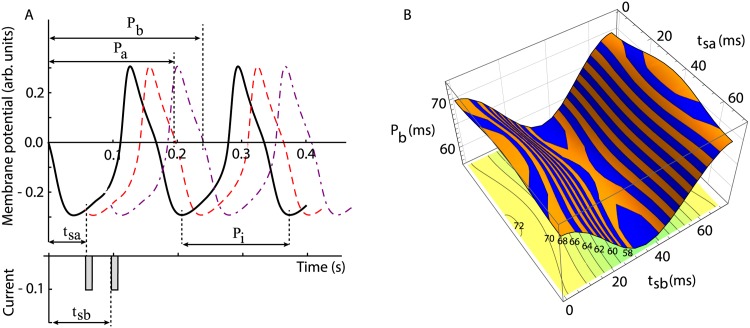
Typical two stimuli PRC. (**A**) Two brief stimuli delivered at stimulus times *t*_*sa*_ and, respectively, *t*_*sb*_. The first stimulus transiently modifies the intrinsic firing period *P*_*i*_ to a new value *P*_*a*_ = *P*_*i*_(1 + *F*^(1)^(*φ*_*a*_)), where *φ*_*a*_ = *t*_*sa*_/*P*_*i*_. The second stimulus arrived at a new phase *φ*_*b*_ = *t*_*ab*_/*P*_*a*_ that found a modified firing period *P*_*a*_ and, therefore, further reset the firing period to *P*_*b*_ = *P*_*a*_(1 + *F*^(1)^(*φ*_*b*_)). (**B**) A typical two stimuli phase response surface for a class I excitable cell.

A typical two stimuli protocol (see Fig 2A) and the corresponding phase resetting *F*^(2)^ are shown in Fig 2B, where the three-dimensional surface is given by Eq (22) in Appendix 1 and a two-dimensional contour plot also shows the contours of equal phase resetting. For this plot we used the analytical normal form of the PRC (see Eq (17) in Appendix 1) where *P*_2*i*_ = 70 ms and the coupling strengths from neuron 1 to neuron 2 was *g*_12_ = 0.015 (excitatory) and from neuron 3 to neuron 2 was *g*_32_ = 0.002 (inhibitory) (see section 3 for a detailed description of the neural model and the synaptic couplings).

## 3 The neural model

In their seminal work on giant squid axon, Hodgkin and Huxley [60–64] experimentally identified three classes, or types, of axonal excitability: class I, where the repetitive firing is controlled by the intensity of an external stimulus; class II, where the firing frequency is almost independent on stimulus intensity; and class III, where there are no endogenous bursters regardless of stimulus intensity or duration.

Our simulations were performed using a class I, single compartment, neural oscillator described by a standard conductance-based, or Hodgkin-Huxley (HH), mathematical model [64–66]. The rate of change of membrane potential is:

$$\begin{array}{ccc} dV/dt & = & -I_{Ca}-I_{K}-I_{Leak}+I_{0}\\ & = & -{\bar{g}}_{Ca}m\left(V\right)\left(V-E_{Ca}\right)-{\bar{g}}_{K}w\left(V-E_{K}\right)-{\bar{g}}_{Leak}\left(V-E_{Leak}\right)+I_{0}, \end{array}$$ (1)

where *V* is the membrane potential, ${\bar{g}}_{ch}$ and *E*_*ch*_ are the maximum conductance and, respectively, the reversal potential for ionic channel *ch* (only calcium, potassium and leak were considered), *w* is the instantaneous probability that a potassium channel is open, and *I*_0_ is a constant bias current. Each ionic current is the product of a voltage-dependent conductance and a driving force *I*_*ch*_ = *g*_*ch*_(*V*)(*V* − *V*_*ch*_) where *g*_*ch*_(*V*) is the product of the maximum conductance for that channel and a specific voltage-dependent gating variable. Morris and Lecar (ML) mathematical model has two non-inactivating voltage-sensitive gating variables: one instantaneous, voltage-dependent, calcium activation *m*(*V*) and a delayed voltage-dependent potassium *w* given by a first order differential equation [67]:

$$\begin{array}{c} dw/dt=\phi (w_{\infty }\left(V\right)-w)/\tau \left(V\right), \end{array}$$ (2)

where *ϕ* is a temperature-dependent parameter, and a voltage-dependent relaxation time constant is defined by *τ*(*V*) = cosh^−1^((*V* − *V*_*w*,1/2_)/(2*V*_*w*,*slope*_)). All open-state probability functions, or steady-state gating variables *x*, have a sigmoidal form [67]:

$$\begin{array}{c} x\left(V\right)=(1+\tanh\left(\left(V-V_{x,1/2}\right)/V_{x,slope}\right))/2, \end{array}$$ (3)

where *V*_*x*,1/2_ is the half-activation voltage and *V*_*x*,*slope*_ is the slope factor for the gating variable *x*. The ML model is widely used in computational neuroscience because it captures relevant biological processes and, at the same time, by changing only a small subset of parameters it can behave either as a type 1 or a type 2 neural oscillator. The dimensionless parameters for a type 1 ML neuron are: *V*_*m*,1/2_ = −0.01, *V*_*m*,*slope*_ = 0.15, *V*_*w*,1/2_ = 0.1, *V*_*w*,*slope*_ = 0.145, *V*_*K*_ = −0.7, *V*_*Leak*_ = −0.5, *V*_*Ca*_ = 1.0, ${\bar{g}}_{Ca}=1.33$, ${\bar{g}}_{K}=2.0$, ${\bar{g}}_{Leak}=0.5$, *I*_0_ = 0.070, and *ϕ* = 0.6 (Ermentrout, 1996). The model’s equations and its parameters are in dimensionless form with all voltages divided by the calcium reversal potential *V*_*Ca*0_ = 120 mV, all conductances divided by ${\bar{g}}_{Ca0}=4$ mS/cm^2^, and all currents normalized by $V_{Ca0}{\bar{g}}_{Ca0}=480\;\mu \text{A}/{\text{cm}}^{2}$ (Ermentrout, 1996). For example, a dimensionless reversal potential for a leak current of *V*_*Leak*_ = −0.5 means *V*_*Leak*_ = −0.5*V*_*Ca*0_ = −0.5 × 120 mV = -60 mV.

**The Synaptic Model.** We implemented fast chemical synapses between neurons given by a synaptic current $I_{syn}={\bar{g}}_{syn}s\left(t\right)\left(V_{post}-E_{syn}\right)$, where ${\bar{g}}_{syn}$ is the maximum synaptic conductance, *s*(*t*) is the fraction of channels activated by neurotransmitters, *V*_*post*_ is the membrane potential of the postsynaptic neuron, and *E*_*syn*_ is the reversal potential of the synaptic coupling. We used *E*_*syn*_ = 0 for excitatory and *E*_*syn*_ = −0.6 for inhibitory coupling. The synapses activation was described by a first order kinetics *s*′ = *αT*(1 − *s*) − *βs*, where *α* = 15, *β* = 1.5, and neurotransmitter binding was described by a sigmoidal function *T*(*V*_*pre*_) = 1/(1 + *e*^(−*V*_*pre*_ − 0.2)120/5)^) where *V*_*pre*_ is the membrane potential of the presynaptic neuron.

**Fig 3:**
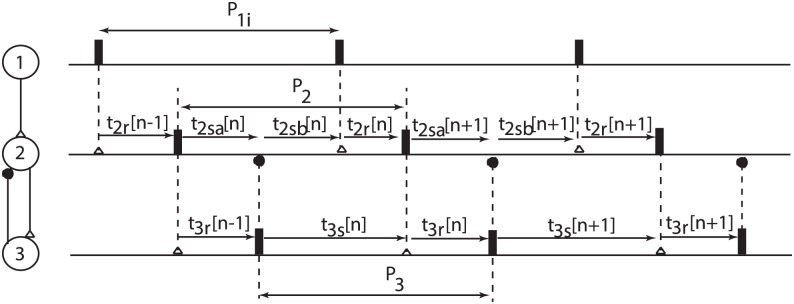
Typical phase-locked mode with one neuron receiving two inputs per cycle. Neuron #1 is the driver of the entire network and its intrinsic firing period *P*_1*i*_ was used as reference duration for all other intrinsic periods. The neuron’s spike is represented by a thick vertical line. The coupling between the neurons is marked by vertical dashed lines that terminate either with an excitatory (empty triangle) or a inhibitory (solid circle) synapse. Neuron #2 receives 2 inputs during one cycle: the first is an inhibition at stimulus time *t*_2*sa*_ from the interneuron #3 and later on it receives an excitatory input from neuron #1 at stimulus time *t*_2*sb*_. The neuron recovers from the last stimulus after *t*_2*r*_ and fires again. Neuron #3 only receives one excitatory input per cycle from neuron #2.

We numerically computed the PRCs in open loop setup, i.e. by injecting a single synaptic input from a corresponding presynaptic neuron for neurons 2 and 3 shown in Fig 3 for our neural network configuration.

## 4 The neural network model

In order to use the PRC method (see section 2) for predicting the relative phases of neurons in a phase-locked firing pattern, we assumed a fixed firing order of the three neurons with the goal of determining if such a pattern exists and if it is stable. Based on the neural network model proposed for delayed and anticipated synchronization by Matias et al [40–42], we identified the following definitions for the firing period of each neuron (see Fig 3):

$$\begin{array}{ccc} P_{1} & = & t_{2r}\left[n-1\right]+t_{2sb}\left[n\right],\\ P_{2} & = & t_{2sb}\left[n\right]+t_{2r}\left[n\right],\\ P_{3} & = & t_{2sb}\left[n\right]-t_{2sa}\left[n\right]+t_{2r}\left[n\right]+t_{2sa}\left[n+1\right], \end{array}$$ (4)

where *t*_2*r*_ is the recovery time of neuron #2 after its last input, *t*_2*sa*_ and *t*_2*sb*_ are the corresponding stimulus times for the first and, respectively, the second input to neuron #2, and the index of the cycle is marked with the square brackets […]. The subscript index refers to the neural oscillator index according to Fig 3. From Eq (4) we eliminated *t*_2*r*_[*n* − 1] = *P*_1*i*_ − *t*_2*sb*_[*n*] and substituted it into the other two equations, which led to:

$$\begin{array}{ccc} P_{2} & = & t_{2sb}\left[n\right]+P_{1i}-t_{2sb}\left[n+1\right],\\ P_{3} & = & t_{2sb}\left[n\right]-t_{2sa}\left[n\right]+P_{1i}-t_{2sb}\left[n+1\right]+t_{2sa}\left[n+1\right]. \end{array}$$ (5)

Based on the definitions of the PRCs (see Eqs (16) and (22) in Appendix 1), we further expanded Eq (5) the transiently modified firing period in terms of experimentally determined PRCs:

$$\begin{array}{ccc} P_{2i}\left(1+F^{\left(2\right)}\left(t_{2sa}\left[n\right],t_{2sb}\left[n\right]\right)\right) & = & t_{2sb}\left[n\right]+P_{1i}-t_{2sb}\left[n+1\right],\\ P_{3i}\left(1+F^{\left(1\right)}\left(t_{s3}\left[n\right]\right)\right) & = & t_{2sb}\left[n\right]-t_{2sa}\left[n\right]+P_{1i}-t_{2sb}\left[n+1\right]+t_{2sa}\left[n+1\right]. \end{array}$$ (6)

The above system of two recursive equations has two unknowns, i.e. *t*_2*sa*_ and *t*_2*sb*_, that describe the temporal evolution of the relative phase of neural oscillators from the firing cycle [*n*] to [*n* + 1].

## 5 The existence of phase-locked modes

Let us assume that there is a steady state solution $\left(t_{2sa}^{*},t_{2sb}^{*}\right)$ for the recursive Eq (6) that mimics the activity of the neural network shown in Fig 3, i.e. the following limits exist $\lim_{n\infty }t_{2sa}\left[n\right]=t_{2sa}^{*}$ and $\lim_{n\infty }t_{2sa}\left[n\right]=t_{2sa}^{*}$. By substituting the steady state, i.e. phase-locked mode, solution $\left(t_{2sa}^{*},t_{2sb}^{*}\right)$ into Eq (6) one obtains:

$$\begin{array}{ccc} P_{2i}\left(1+F_{2}^{\left(2\right)}\left(t_{2sa}^{*},t_{2sb}^{*}\right)\right) & = & P_{1i},\\ P_{3i}\left(1+F_{3}^{\left(1\right)}\left(P_{1i}-t_{2sa}^{*}\right)\right) & = & P_{1i}, \end{array}$$ (7)

where we used the fact that $t_{3s}^{*}=t_{2sb}^{*}-t_{2sa}^{*}+t_{2r}^{*}$ and that *t*_2*r*_[*n* − 1] = *P*_1*i*_ − *t*_2*sb*_[*n*], which led to $t_{3s}^{*}=t_{2sb}^{*}-t_{2sa}^{*}+P_{1i}-t_{2sb}^{*}=P_{1i}-t_{2sa}^{*}.$

As we notice from the second equation in Eq (7), the steady state value $t_{2sa}^{*}$ could be immediately determined and it only depends on *P*_1*i*_, *P*_3*i*_, and the PRC of the third neuron, which depends on the coupling strength *g*_23_. It results that the steady state value $t_{2sa}^{*}$ is given by:

$$\begin{array}{c} F_{3}^{\left(1\right)}\left(P_{1i}-t_{2sa}^{*}\right)=\frac{P_{1i}}{P_{3i}}-1. \end{array}$$ (8)

Since the coupling from neuron #2 to neuron #3 is excitatory, the PRC is negative (only advances the next spike), i.e. $F_{3}^{\left(1\right)}\left(\varphi \right)<0$. As a result of Eq (8), the steady states $t_{2sa}^{*}$ can only exist for *P*_1*i*_ < *P*_3*i*_ which means that the interneuron (neuron #3) must be slower than the pacemaker (neuron #1) of the network. Moreover, since a type 1 PRC in response to excitatory inputs has only one negative minimum ($F_{3,min}^{\left(1\right)}$) that determines the magnitude of the strongest possible resetting, then $\frac{P_{1i}}{P_{3i}}-1\geq F_{3,min}^{\left(1\right)}$, which means the the interneuron intrinsic period is bounded by $P_{1i}<P_{3i}<P_{1i}/\left(1+F_{3,min}^{\left(1\right)}\right).$

Once we determined the steady state $t_{2sa}^{*}$ from Eq (8), then we plugged it into the first Eq (7) and found $t_{2sb}^{*}.$ Using the PRC definition (see Eq (22) from Appendix 1) we obtained:

$$\begin{array}{c} P_{2i}\alpha \left(1+F_{2}^{\left(1\right)}\left(\frac{t_{2sb}^{*}}{\alpha }\right)\right)=P_{1i}, \end{array}$$ (9)

where $\alpha =1+F_{2}^{\left(1\right)}\left(t_{2sa}^{*}\right).$ The above equation can be reduced to:

$$\begin{array}{c} F_{2}^{\left(1\right)}\left(\frac{t_{2sb}^{*}}{\alpha }\right)=\frac{P_{1i}}{P_{2i}\alpha }-1, \end{array}$$ (10)

We must emphasize that $F_{2}^{\left(1\right)}\left(t_{2sa}^{*}\right)$ and $F_{2}^{\left(1\right)}\left(t_{2sb}^{*}\right)$ are two different single stimulus PRCs for the same neuron. Here $F_{2}^{\left(1\right)}\left(t_{2sa}^{*}\right)$ is the single stimulus phase response curve of the second neuron to an input received from the third neuron, i.e. $F_{2}^{\left(1\right)}\left(t_{2sa}^{*}\right)$ is determined by *g*_32_. Similarly, $F_{2}^{\left(1\right)}\left(t_{2sb}^{*}\right)$ is the single stimulus phase response curve of the second neuron in response to an input received from the first neuron, i.e. $F_{2}^{\left(1\right)}\left(t_{2sb}^{*}\right)$ is determined by *g*_12_.

### 5.1 Explicit steady state solutions using normal form generic type 1 PRCs

In order to get insights into the general existence criteria for steady state (phase-locked modes) derived above, we assumed that the single stimulus and the generalized PRCs are quite well approximated by the corresponding normal forms given by Eq (16) and, respectively, by Eq (22) in Appendix 1. Then the steady state solution $t_{2sa}^{*}$ of Eq (8) can be analytically written as:

$$\begin{array}{c} \cos\left(2\pi \left(\frac{P_{1i}}{P_{3i}}-\frac{t_{2sa}^{*}}{P_{3i}}\right)\right)=1-\frac{1}{c_{23}P_{3i}}\left(\frac{P_{1i}}{P_{3i}}-1\right). \end{array}$$ (11)

By least square fitting the numerically generated PRCs for each neuron in response to a single spike from its corresponding presynaptic neuron with the theoretical formula of the normal form given by Eq (17), we found a quantitative relationship between the abstract coupling strength coefficient *c* and the physiologically measurable maximum synaptic couplings $\bar{g}$ (see Appendix 1). Therefore, in order to simplify the mathematical notation, throughout the rest of the paper we only write, for example, *c*_23_ when referring to the coefficient of the theoretical normal form of the PRC with the understanding that it is a known function of the synaptic conductance, i.e. *c*_23_ = *c*_23_(*g*_23_).

**Fig 4:**
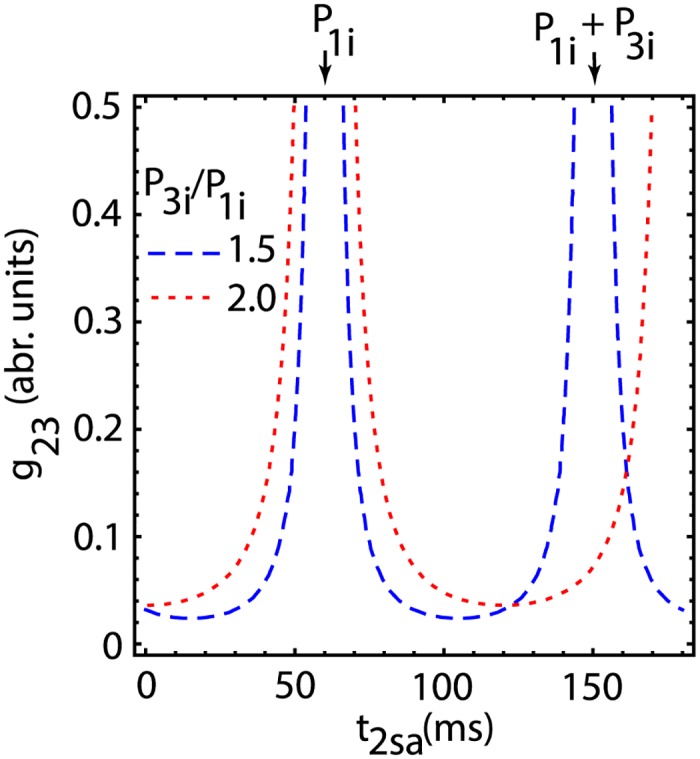
Minimum coupling strength *g*_23_ required for a given phase-locked mode time $t_{2sa}^{*}$. (a) There are multiple possible solutions for $t_{2sa}^{*}$ for the same coupling strength between neurons #2 and #3 due to the PRC periodicity. In the limit case of a very strong coupling (*g*_23_∞) the phase-locked stimulus time becomes $t_{2sa}^{*}=P_{1i}+kP_{3i}$ (see the vertically downward arrows).

Since −1 ≤ *cos*(*x*)≤1, it results that $0\leq \frac{1}{c_{23}\left(g_{23}\right)P_{3i}}\left(\frac{P_{1i}}{P_{3i}}-1\right)\leq 2$, which determines the minimum coupling strength *g*_23_ for a given ratio of the two intrinsic firing periods $\frac{P_{1i}}{P_{3i}}$ to attain a phase-locked mode pattern. Based on the above relationship, for excitatory coupling, i.e. *E*_*syn*_ = 0, the master (pacemaker) neuron #1 (see Fig 3) must be faster than the interneuron #3, i.e. *P*_1*i*_ < *P*_3*i*_. At the same time, the coupling strength *g*_23_ must also be strong enough to reset the longer intrinsic period *P*_3*i*_ to match the shorter period of the network’s pacemaker, i.e. to ensure that $P_{3i}<P_{1i}/\left(1+F_{3,min}^{\left(1\right)}\right)$. The above relationship allowed us to estimate that, if the coupling is very strong (*g*_23_∞), then the steady state from Eq (11) has the solution $t_{2sa}^{*}=P_{1i}+kP_{3i}$ with *k* = 0,±1,±2,…, which is marked by vertically downward arrows in Fig 4. Furthermore, if the two firing periods are approximately equal (*P*_1*i*_ ≈ *P*_3*i*_), then from Eq (11) it results that $t_{2sa}^{*}=kP_{1i}$ with *k* = 0, 1, 2, … (see also Fig 4). Fig 4 also shows that for each intrinsic period ratio *P*_3*i*_/*P*_1*i*_ there is a minimum coupling strength *g*_23_ that ensures appropriate resetting of the interneuron. For example, the minimum coupling for *P*_3*i*_/*P*_1*i*_ = 1.5 (dotted red line in Fig 4) is *g*_23_ = 0.024. A stronger coupling of *g*_23_ = 0.036 is necessary for a larger ratio *P*_3*i*_/*P*_1*i*_ = 2 (dashed blue line in Fig 4)).

The phase-locked modes $\left(t_{2sa}^{*},t_{2sb}^{*}\right)$ given by Eq (6) depend on three intrinsic periods *P*_1*i*_, *P*_2*i*_, *P*_3*i*_ and three synaptic conductances *g*_12_, *g*_23_, and *g*_32_. Since the master neuron receives no input, all durations were measured relative to *P*_1*i*_. The bias current for the computational model was set such that *P*_1*i*_ = 60 ms, *P*_2*i*_ = 70 ms and *P*_3*i*_ = 80 ms (see section 3 for details and supplemental files for a computational implementation). Intuitively, the phase-locked solution $t_{2sa}^{*}$ is the stable interspike interval between neurons #2 and #3 (the interneuron). The other phase-locked solution $t_{2sb}^{*}$ is the stable interspike interval between neurons #2 and #1 (network’s driver). However, this simplification only reduces the parameter space to five dimensions.

**Fig 5:**
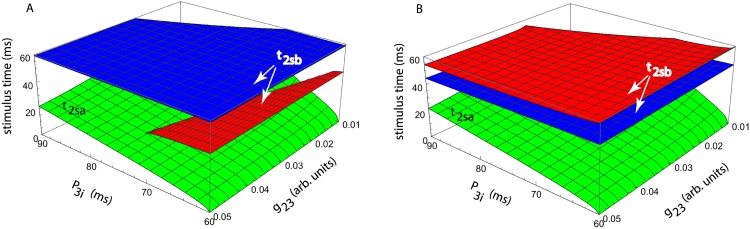
Phase-locked solutions $t_{2sa}^{*}$ and $t_{2sb}^{*}$ (vertical axes) versus the intrinsic period of the interneuron *P*_3*i*_ and the coupling strength *g*_23_. (**A**) The first stimulus time $t_{2sa}^{*}$ only depends on *P*_3*i*_/*P*_1*i*_ and *g*_23_—see green surface. For a fixed intrinsic period ratio *P*_2*i*_/*P*_1*i*_ = 70/60, the second stimulus time $t_{2sb}^{*}$ depends also on the coupling strength *g*_12_ = 0.012 (red surface) and *g*_12_ = 0.05 (blue surface). (**B**) For a fixed coupling *g*_12_ = 0.015, the second stimulus time $t_{2sb}^{*}$ dependence on the intrinsic firing periods *P*_2*i*_/*P*_1*i*_ = 60/60 (red surface) and *P*_2*i*_/*P*_1*i*_ = 70/60 (blue surface) shows that the solution space is wider for shorter intrinsic periods.

In order to reduce the parameter space to four dimensions, we only show examples of phase-locked modes for a fixed inhibitory coupling with *g*_32_ = 0.002 (arb. units). For a fixed intrinsic period of the second neuron *P*_2*i*_/*P*_1*i*_ = 70/60, the parameter space further reduces to three dimensions, which allowed us to visualize the phase-locked modes. The solution $t_{2sa}^{*}$ of the second equation in Eq (6) only depends on *P*_3*i*_/*P*_1*i*_ and the coupling strength *g*_23_ (see the green surface in Fig 5).

However, the phase locked solution $t_{2sb}^{*}$ of the first equation in Eq (6) depend on the additional coupling *g*_12_. Therefore, to gain insight into how *g*_12_ affects the solution $t_{2sb}^{*}$, we used the same axis *P*_3*i*_/*P*_1*i*_ and *g*_23_ as for $t_{2sa}^{*}$, but with different constant values of coupling *g*_12_ = 0.012 (red surface) and *g*_12_ = 0.05 (blue surface) in Fig 5A. We notice from Fig 5A that increasing the strength of the excitatory coupling *g*_12_ leads to an increased stimulus time $t_{2sb}^{*}$ and a wider parameter domain of the phase-locked solution.

Similarly, if we hold constant the synaptic coupling *g*_12_ = 0.015 (arb. units) between the master and the slave neurons, then we could visualize the phase-locked solution for variable intrinsic period of the second neuron *P*_2*i*_/*P*_1*i*_ = 60/60 (red surface in Fig 5B) and *P*_2*i*_/*P*_1*i*_ = 70/60 (blue surface in Fig 5B). We notice that for smaller intrinsic periods *P*_2*i*_ the range of control parameters *P*_3*i*_/*P*_1*i*_ and *g*_23_ is broader. This is because for more similar firing frequencies it is easier to bring the driven neuron to the firing frequency of the driving neuron.

## 6 The stability of phase-locked modes

The possible phase-locked modes given by Eq (6) may not all be stable and, therefore, they may not be all experimentally observable. To determine the stability of the steady solutions $\left(t_{2sa}^{*},t_{2sb}^{*}\right)$, we assume small perturbations:

$$\begin{array}{ccc} t_{2sa}\left[n\right] & = & t_{2sa}^{*}+\delta t_{2sa}\left[n\right],\\ t_{2sb}\left[n\right] & = & t_{2sb}^{*}+\delta t_{2sb}\left[n\right], \end{array}$$ (12)

where the *n*^*th*^ cycle perturbation $\delta t_{2s}\left[n\right]<<t_{2s}^{*}$ is assumed very small for both stimuli. By substituting Eq (12) into the existence criteria from Eq (7) and using a Taylor series expansion one obtains:

$$\begin{array}{ccc} m_{2a}\left(1+F_{2}^{\left(1\right)}\left(\varphi_{2b}^{*}\right)-\varphi_{2b}^{*}\right)\delta t_{2sa}\left[n\right]+m_{2b}\delta t_{2sb}\left[n\right] & = & \delta t_{2sb}\left[n\right]-\delta t_{2sb}\left[n+1\right],\\ m_{3}\left(\delta t_{2sb}\left[n\right]-\delta t_{2sa}\left[n\right]-\delta t_{2sb}\left[n+1\right]\right) & = & \delta t_{2sb}\left[n\right]-\delta t_{2sa}\left[n\right]\\ & = & -\delta t_{2sb}\left[n+1\right]+\delta t_{2sa}\left[n+1\right], \end{array}$$ (13)

where $m_{2a}={\left(\frac{\partial F_{2}^{\left(1\right)}}{\partial \varphi }\right)}_{\varphi_{2a}^{*}}$ is the slope of the second neuron’s PRC at the phase of the first stimulus $\varphi_{2a}^{*}=\frac{t_{s2a}^{*}}{P_{2i}}$, $m_{2b}={\left(\frac{\partial F_{2}^{\left(1\right)}}{\partial \varphi }\right)}_{\varphi_{2b}^{*}}$ is the slope of the second neuron’s PRC at the phase of the second stimulus $\varphi_{2b}^{*}=\frac{t_{s2b}^{*}}{P_{2i}\left(1+F_{2}^{\left(1\right)}\left(t_{2sa}^{*}\right)\right)}$, $m_{3}={\left(\frac{\partial F_{3}^{\left(1\right)}}{\partial \varphi }\right)}_{\varphi_{3}^{*}}$ is the slope of the third neuron’s PRC at the phase of the stimulus $\varphi_{2}^{*}=\frac{t_{s3}^{*}}{P_{3i}}$. The stability Eq (13) can be rewritten in a matrix form as:

$$\begin{array}{c} \left(\begin{array}{cc} 0 & -1\\ 1 & m_{3}-1 \end{array}\right){\left(\begin{array}{c} \delta t_{s2a}\\ \delta t_{s2b} \end{array}\right)}_{\left[n+1\right]}=\left(\begin{array}{cc} m_{2a}\left(1+F_{2}^{\left(1\right)}\left(\varphi_{2b}^{*}\right)-\varphi_{2b}^{*}\right) & m_{2b}-1\\ 1-m_{3} & m_{3}-1 \end{array}\right){\left(\begin{array}{c} \delta t_{s2a}\\ \delta t_{s2b} \end{array}\right)}_{\left[n\right]}, \end{array}$$ (14)

which led us to a first order recursive relationship for the perturbations:

$$\begin{array}{c} {\left(\begin{array}{c} \delta t_{s2a}\\ \delta t_{s2b} \end{array}\right)}_{\left[n+1\right]}=\left(\begin{array}{cc} a_{11} & a_{12}\\ a_{21} & a_{22} \end{array}\right){\left(\begin{array}{c} \delta t_{s2a}\\ \delta t_{s2b} \end{array}\right)}_{\left[n\right]}, \end{array}$$ (15)

where *a*_11_ = (1 − *m*_3_)(1 − *b*), *a*_12_ = (*m*_3_ − 1)*m*_2*b*_, *a*_21_ = −*b*, and *a*_22_ = 1 − *m*_2*b*_ with $b=m_{2a}\left(1+F_{2}^{\left(1\right)}\left(\varphi_{2b}^{*}\right)-m_{2b}\varphi_{2b}^{*}\right).$ The stability of the steady state is determined by the eigenvalues of Eq (15) (see the Appendix 2 for the general stability conditions in a two-dimensional recursive map).

We also must keep track of the third stability condition as the original recursive system in Eq (4) contained three variables, which were reduced to two coupled recursive equations (see Eq (5)) by eliminating the third variable, i.e. *t*_2*r*_[*n* − 1] = *P*_1*i*_ − *t*_2*sb*_[*n*]. As a result, the steady state of the previous substitution gives $t_{2r}^{*}=P_{1i}-t_{2sb}^{*}$ and the corresponding infinitesimal perturbation is *δt*_2*r*_[*n* − 1] = −*δt*_2*sb*_[*n*]. Therefore, the stability of $t_{2r}^{*}$ solution is determined by the stability of *δt*_2*sb*_[*n*], which is already covered by Eq (15) without involving additional control parameters.

The general stability conditions for any first order recursion of two variables is discussed in details in the Appendix 2. Briefly, the trace *Tr*(*A*) = *a*_11_ + *a*_22_ and the determinant *Det*(*A*) = *a*_11_ *a*_22_ − *a*_12_ *a*_21_ of the recursion matrix in Eq (15) determine the stability of each steady state obtained by solving Eq (7).

## 7 Numerical validation of the existence and the stability criteria

The analytically derived criteria for the existence (see section 5) and stability (see section 6) of phase-locked modes in a master-slave network with a dynamic loop (see Fig 3) were only based on PRCs in response to a single stimulus. We checked our theoretical predictions based on open loop PRCs against the numerical simulations of the actual neural networks implemented according to the model presented in section 3, i.e. closed loop (fully connected neural network).

**Fig 6:**
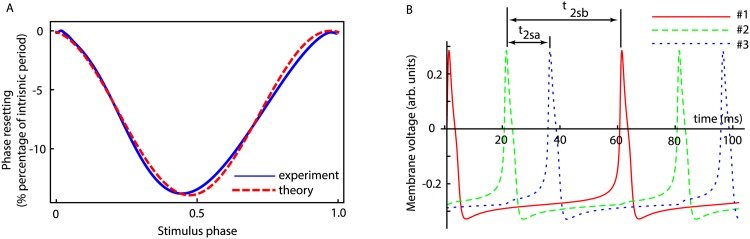
Phase-locked modes in fully coupled neural network. (**A**) The numerically generated PRC in open loop setup in response to a single triangularly shaped stimulus (solid circles) was fitted to the theoretical PRC given by Eq (17) to determined the conversion factor between the model-dependent coupling constant *c* in Eq (17) and the synaptic coupling *g*_*syn*_. (**B**) A typical stable phase locked mode in which neuron #2 (dashed line) receives two inputs during a single cycle: first from neuron #3 (dotted line) at $t_{2sa}^{*}$ and then from neuron #1 (continuous line) at $t_{2sb}^{*}$. The experimental values for the phase locked mode as measured from panel (**B**) were $t_{2sa}^{*}=15.2$ ms and $t_{2sb}^{*}=40.2$ ms whereas the PRC-based predictions were $t_{2sa}^{*}=18.0$ ms and $t_{2sb}^{*}=44.1$ ms. The network’s firing period was *P* = 60 ms = *P*_1*i*_.

The analytical normal form PRC formulas (see Eq (17) in Appendix 1) were convenient analytical tools and even led us to some analytical results in the preceding sections. However, for the actual comparison between the multiple stimuli PRC-based phase-locked mode prediction (open loop) and the numerical simulations results of the fully coupled neural network (see Fig 3) we used numerically generated open lopp PRCs. The reason is that, although the analytical normal form of type 1 PRC given by Eq (17) (see dashed red line in Fig 6A) is close to the numerically (experimentally) generated open loop PRC (see dotted blue curve in Fig 6A), we wanted a more accurate prediction based on the real-world PRC as it is generated in wet lab/numerical experiments.

We also used the least square minimization to fit actual PRCs (see dotted curve in Fig 6A) with the theoretical formula given by Eq (17) in order to establish the conversion factor between the model-dependent coupling constant *c* in the theoretical formula given by Eq (17) and the synaptic constant *g*_*syn*_ used in our numerical simulations. The Mathematica file that contains the implementation of the neural network shown in Fig 3 based on the model equations provided in section 3 is available in supplemental files section.

The synaptic couplings used for the example shown in Fig 6B were *g*_12_ = 0.015, *g*_32_ = 0.002, and *g*_23_ = 0.0275, which led to a phase locked mode with $t_{2sa}^{*}=15.2$ ms and $t_{2sb}^{*}=40.2$ ms. The PRC-based predictions were $t_{2sa}^{*}=18.0$ ms (about 18% error) and $t_{2sb}^{*}=44.1$ ms (about 10% error). We found that the eigenvalues of the stability matrix were *λ*_1_ = 0.489, and *λ*_2_ = 0.779, which indicated that the predicted mode was stable.

## Discussion

Since even for a small unidirectionally coupled three-neuron network the parameter space is six-dimensional, i.e. three intrinsic firing periods (*P*_1*i*_, *P*_2*i*_, and *P*_3*i*_), one unidirectional synaptic coupling between master-slave neurons (*g*_12_), and two coupling constants for the feedback loop (*g*_23_ and *g*_32_), we reduced it to manageable dimensions in order to visualize the phase-locked solution. Since the master neuron receives no feedback from the network, its intrinsic firing period *P*_1*i*_ was considered the reference duration, which reduces the parameter space to five dimensions. We further reduced the parameter space to four dimensions using a fixed value for the inhibitory coupling of the interneuron, i.e. *g*_32_ = 0.002 (arb. units). We numerically found the phase locked modes $\left(t_{2sa}^{*},t_{2sb}^{*}\right)$ by considering two separate cases: (1) fixed period of slave neuron #2, of which we only show two examples with *P*_2*i*_/*P*_1*i*_ = 60/60 and *P*_2*i*_/*P*_1*i*_ = 70/60 in Fig 5A and (2) fixed master-slave synaptic coupling, of which we only showed two examples with *g*_12_ = 0.012 and *g*_12_ = 0.05 in Fig 5B. In all numerical simulations, the free parameters were the intrinsic period of the interneuron *P*_3*i*_ and the excitatory synaptic coupling to that neuron (*g*_23_). The reason is that it was previously shown that the interneuron through its intrinsic properties and its synaptic coupling can lead to either delayed or anticipating synchronization in this neural network [40, 42] and our goal was to closely match previous experimental findings using the newly developed generalized PRC method. Based on Fig 5, an increase in the strength of the master-slave synaptic coupling *g*_12_ leads to a larger phase difference between the two steady states $t_{2sa}^{*}$ and $t_{2sb}^{*}$. At the same time, the parameter space of the interneuron (*P*_3*i*_, *g*_22_) becomes wider. Another possibility for broadening the parameter space was to bring the intrinsic firing period of the slave neuron #2 closer the the master neuron #1, i.e. by reducing network heterogeneity. All out numerical simulations are in agreement with previously observed firing patterns in this type of neural network [40, 42].

## Conclusions

We used a phase response curve method to predict the existence and the stability of phase-locked modes in a master-slave networks with a dynamic feedback loop. This study brings two novel solutions to phase-locked mode prediction in neural networks. First, we generalized the the phase response curve definition to include the more realistic case when neural oscillators receive more than one input per cycle. Secondly, we applied the generalized phase resetting definition to a biologically relevant neural network that has been shown to produce both delayed and anticipated synchronization.

Predicting phase-locked modes in large neural networks usually requires as a first step a complexity reduction to manageable subnetworks of two neurons [68, 69] or, whenever possible, reduces the entire network to a two-population network [70]. Our PRC generalization to multiple inputs per cycle is a significant advance in phase resetting theory that allows investigation of large networks in which individual neurons receive multiple inputs per cycle without assuming special network connectivity. Furthermore, our generalization of phase response curve and its proof of concept application to predicting phase-locked modes existence and stability in a biologically relevant three-neuron network with a dynamic feedback loop is not limited to weak coupling nor to only one-to-one firing patterns. Indeed, the coupling strengths used were quite large such that it reset the firing period of the interneuron #3 by 25% from 80 ms to 60 ms.

## Appendix 1

### Single stimulus phase response curve method

There are two main experimental protocols for measuring the single stimulus PRC in isolated cells: (1) single stimulus and (2) recurring (periodic) stimuli. In the case of a single stimulus protocol, a free running neural oscillator with the intrinsic period *P*_*i*_ is perturbed at a certain instant called stimulus time *t*_*s*_, which is measured from an arbitrary phase reference *φ* = 0, e.g. zero crossing of the membrane potential with a positive slope. As a result of the perturbation, the length of the current cycle that contains the stimulation (see Fig 1A) may be transiently shortened or lengthened to a new duration *P*_1_. The relative change in the duration of the current cycle with respect to the unperturbed duration *P*_*i*_ determines the first order PRC in response to a single and nonrecurring stimulus:

$$\begin{array}{c} F^{\left(1\right)}\left(\varphi \right)=P_{1}/P_{i}-1, \end{array}$$ (16)

where the superscript ^(1)^ emphasizes that the resetting is due to a single input per cycle, which has been used as the “classical” definition of PRC. Based on Eq (16), a negative value of the PRC means that the next spike is advanced, otherwise it is delayed. Others [58, 71] prefer to flip the sign in Eq (16) and associate a positive sign to a phase advance. Oftentimes, the effect of a single stimulus extends to subsequent cycles and is measured by higher order PRCs [47, 48, 50]. Usually, one records at least five cycles until the neural oscillatory returns back to its unperturbed oscillatory activity [31, 32]. Afterwards another single stimulus is applied at a different phase to quantify its effect on the isolated neuron (open loop experimental setup).

In the case of recurring external stimuli, the interpretation of the phase resetting and its usage in phase-locked mode prediction is complicated by (1) the fact that the measured resetting compounds multiple PRC orders in a potentially nonlinear manner and (2) the activation of slow currents and/or long term potentiation (see [72] for examples and [32] for higher order PRC applications).

**Normal Forms of Single Stimulus Phase Response** Curves. A saddle-node bifurcation, which presents a continuous frequency versus bias current (f-I) curve that extends to arbitrarily low frequencies (see solid circles in Fig 1B) usually leads to a type 1 PRC that looks unimodal as in Fig 1C (although for counterexamples see [49, 56]). Close to the bifurcation point, type 1 unimodal PRCs are described analytically by the following equation [57]:

$$\begin{array}{c} F^{\left(1\right)}\left(\varphi \right)=\frac{c_{SN}}{\omega }\left(1-\cos\left(2\pi \varphi \right)\right), \end{array}$$ (17)

where *c*_*SN*_ is a constant determined by the neural model and *ω* = 2*π*/*P*_*i*_ is the intrinsic angular frequency of the oscillator. In this study, we used the simplified analytical form given by Eq (17) to get analytical insights into the general behavior of the three-neuron network with a dynamic loop shown in Fig 3.

By least square fitting the numerically generated PRCs for each neuron in response to a single spike from its corresponding presynaptic neuron with the theoretical formula of the normal form PRC given by Eq (17), we found coupling strengths *c* are proportional to the maximum synaptic couplings $\bar{g}$: *c*_12_ = −6.1733*g*_12_ − 0.0003, *c*_23_ = −6.9555*g*_23_ − 0.0005, and *c*_32_ = 7.2764*g*_32_ + 0.0002.

### Phase resetting in response to multiple stimuli

Assuming that the resetting induced by one stimulus takes effect “almost” instantaneously, i.e. before the arrival of the second stimulus, then the effects of two stimuli applied during the same cycle are independent of each other and we could use the single stimulus PRC defined by Eq (16) (shown in Fig 1C and 1D) to compute the phase resetting in response to two or more stimuli. In order to compute the phase resetting induced by the second stimulus based on Eq (16) we need to correctly compute its phase (see Fig 2A). The phase of the first stimulus that arrives at a stimulus time *t*_*sa*_ is *φ*_*a*_ = *t*_*sa*_/*P*_*i*_. The first stimulus produces an “almost” instantaneously phase resetting and changes the firing period to:

$$\begin{array}{c} P_{a}=P_{i}\left(1+F^{\left(1\right)}\left(\varphi_{a}\right)\right)=P_{i}\left(1+F^{\left(1\right)}\left(\frac{t_{a}}{P_{i}}\right)\right). \end{array}$$ (18)

When the second stimulus arrives at a stimulus time *t*_*sb*_ > *t*_*sa*_, the neuron already has a different firing period *P*_*a*_ due to the previous stimulus. As a result, the phase of the second stimulus is *φ*_*b*_ = *t*_*sb*_/*P*_*a*_ and the new firing period due to the second stimulus is:

$$\begin{array}{c} P_{b}=P_{a}\left(1+F^{\left(1\right)}\left(\varphi_{b}\right)\right)=P_{a}\left(1+F^{\left(1\right)}\left(\frac{t_{b}}{P_{a}}\right)\right), \end{array}$$ (19)

where we used the same definition of the first order phase resetting for a single stimulus as in Eq (16). By substituting Eq (18) into Eq (19) one obtains:

$$\begin{array}{c} P_{b}=P_{a}\left(1+F^{\left(1\right)}\left(\varphi_{b}\right)\right)=P_{i}\left(1+F^{\left(1\right)}\left(\frac{t_{a}}{P_{i}}\right)\right)\left(1+F^{\left(1\right)}\left(\frac{t_{b}}{P_{i}\left(1+F^{\left(1\right)}\left(\frac{t_{a}}{P_{i}}\right)\right)}\right)\right), \end{array}$$ (20)

which could be rewritten in a form that resembles Eq (16) as:

$$\begin{array}{c} P_{b}=P_{i}\left(1+F^{\left(2\right)}\left(\varphi_{a},\varphi_{b}\right)\right), \end{array}$$ (21)

where the superscript ^(2)^ emphasizes that the new transient period *P*_*b*_ is computed in response to two stimuli arriving at phases *φ*_*a*_ and, respectively, *φ*_*b*_ > *φ*_*a*_ during the same cycle. By comparing the definition from Eq (21) against the derived resetting from Eq (20), we found that:

$$\begin{array}{c} F^{\left(2\right)}\left(t_{sa},t_{sb}\right)=\left(1+F^{\left(1\right)}\left(\frac{t_{a}}{P_{i}}\right)\right)\left(1+F^{\left(1\right)}\left(\frac{t_{b}}{P_{i}\left(1+F^{\left(1\right)}\left(\frac{t_{a}}{P_{i}}\right)\right)}\right)\right)-1, \end{array}$$ (22)

which has the advantage that can predict the phase resetting in response to two stimuli by recursively using the single stimulus PRC defined in Eq (16). A typical two stimuli phase response curve *F*^(2)^ is shown in Fig 2B.

Furthermore, our novel derivation of PRC in response to two stimuli given by Eq (22) generalizes to an arbitrary number of inputs per cycle as follows:

$$\begin{array}{c} P_{n}\left(t_{s1},t_{s2},\ldots ,t_{sn}\right)=P_{i}\prod_{k=1}^{n}\left(1+F^{\left(1\right)}\left(\frac{t_{sk}}{P_{k-1}}\right)\right), \end{array}$$ (23)

where *P*_0_ = *P*_*i*_ is the intrinsic firing period of the isolated neuron, *t*_*sk*_ > *t*_*s*(*k* + 1)_, and *t*_*sk*_ < *P*_*k*−1_ (stimulus *k* still falls inside the transiently modified period due to the previous stimulus).

## Appendix 2

**Stability Conditions for Two-Dimensional Recursive Maps.** The characteristic polynomial of any first order recursive equation of two variables, such as Eq (6), is:

$$\begin{array}{c} P\left(\lambda \right)=\lambda^{2}-\left(Tr\left(A\right)\right)\lambda +Det\left(A\right), \end{array}$$ (24)

where Tr(A) and Det(A) are the trace and, respectively, the determinant of the recursion matrix of the perturbations (*δt*_2*sa*_, *δt*_2*sb*_), such as the Eq (15). The first order recursions have the following solution:

$$\begin{array}{c} \delta t\left[n\right]=C_{1}\lambda_{1}^{n}+C_{2}\lambda_{2}^{n}, \end{array}$$ (25)

where *C*_1_ and *C*_2_ are some constants determined from the initial conditions and *λ*_*i*_ (with *i* = 1, 2) are the solutions of the characteristic polynomial Eq (24). For the perturbations to die out, all characteristic roots must be less than unit, i.e. |*λ*_*i*_|<1 for both *i* = 1, 2. To ensure stability, there are two possibilities: (1) the roots of the characteristic polynomial are real and both less than the unit, or (2) the roots are complex conjugated with a magnitude less than the unit.

**Real characteristic roots.** In this case, the following conditions must be met

$$\begin{array}{ccc} P(-1)=1+Tr(A)+Det(A) & > & 0,\\ P(+1)=1-Tr(A)+Det(A) & > & 0,\\ Det\left(A\right)-{\left(Tr(A)\right)}^{2}/4 & > & 0. \end{array}$$ (26)

**Fig 7:**
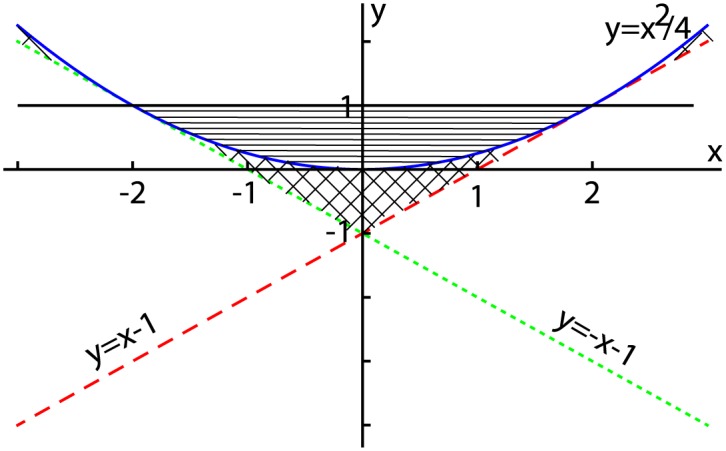
Stability regions of the two-dimensional recursive maps. The stability condition for any recursive maps requires that all roots of the characteristic polynomial are less than unit (|*λ*| < 1). For a two-dimensional, first order, recursive there are only two parameters that control the stability conditions above, i.e. the trace *x* = *Tr*(*A*) and the determinant *y* = *Det*(*A*) of the characteristic matrix. The parabolic curve in (*x*, *y*) plane separates real from imaginary roots of characteristic polynomial. For real roots, i.e. below the parabolic curve, the stability region is only limited to the areas above the two tangent lines to the parabola (see 45 degree hashed areas). For imaginary roots, i.e. above the parabolic curve, the stability region is also limited to the area below the unit value since $\left|\lambda \right|=\sqrt{y}<1$ (see hashed area with horizontal lines).

The region where all three conditions are met is shown in Fig 7 with crossed hashing, i.e. the region below the parabolic curve and above the two straight, tangent, lines.

**Imaginary characteristic roots.** In this case, the discriminant of the characteristic polynomial is negative, i.e. −*Det*(*A*) + (*Tr*(*A*))^2^/4 < 0. At the same time, the magnitude of each complex conjugated characteristic root is $\left|\lambda \right|=\sqrt{Det(A)}<1$, i.e. *Det*(*A*) < 1. As a result, the stability region in the case of complex characteristic root is above the parabolic shape shaded with horizontal lines in Fig 7.

## Supporting information

S1 File Mathematica code. The Mathematica code simulates the driven-driver neural network with adaptive feedback. It uses Morris-Lecar type 1 neurons and chemical couplings between neurons to produce a stable phase-locked firing pattern.
(NB) Click here for additional data file.
